# Glomérulonéphrite extra-membraneuse et syndrome myélodysplasique: une association rare

**DOI:** 10.11604/pamj.2018.29.85.3486

**Published:** 2018-01-30

**Authors:** Mahtat El Mehdi, Ahmed Alayoude, Mohamed Amine Hamzi, Wafe Arache, Kawtar Hassani, Selim Jennane, Hicham Eddou, Kamal Doghmi, Mohamed Mikdame

**Affiliations:** 1 Service d’Hématologie Clinique, Hôpital Militaire d’Instruction Mohamed V, Rabat, Maroc; 2 Service de Néphrologie et de Transplantation Rénale, Hôpital Militaire d’Instruction Mohamed V, Rabat, Maroc

**Keywords:** Glomérulonéphrite extra-membraneuse, syndrome myélodysplasique, Maroc, Extramembranous glomerulonephritis, myelodysplastic syndrome, Morocco

## Abstract

Les syndromes myélodysplasiques peuvent s’accompagner de maladies auto-immunes. L’atteinte rénale au cours de ces syndromes est rare. Dans ce cas, les glomérulopathies prédominent cette atteinte. La glomérulonéphrite extra-membraneuse est exceptionnellement reportée en association avec un syndrome myélodysplasique. Nous rapportons dans ce papier le cas d’une patiente présentant une glomérulonéphrite associée à une anémie révélant un syndrome myélodysplasique de faible risque. Dans la lumière de ce cas, nous faisons une courte revue de la littérature des cas précédemment publiés et nous discutons le lien pathogénique entre ces deux entités.

## Abstract (en)

Myelodysplastic syndromes may be associated with autoimmune diseases. Renal involvement is rare but, if occurs, it manifests predominantly as glomerular diseases. Extramembranous glomerulonephritis associated with myelodysplastic syndrome has been reported very rarely. We here report the case of a patient presenting with glomerulonephritis associated with anemia, revealing low-risk myelodysplastic syndrome. In the light of this case, we conducted a review of the literature of previously published cases and discussed the pathogenic link between these two entities.

## Introduction

Il est reporté que 10 à 20% des patients atteints de syndrome myélodysplasique (SMD) présentent des maladies auto-immunes (MAI) [1]. L’incidence du syndrome néphrotique est plus élevée chez ces patients par rapport à la population générale [2]. L’association d’une glomérulonéphrite extra-membraneuse (GEM) et d’un SMD chez l’adulte est exceptionnelle, peu de cas ont été rapportés dans la littérature [3]. Nous décrivons dans ce case-report l’observation d’une patiente présentant un SMD de faible risque associé à une GEM.

## Patient et observation

**Figure 1:**
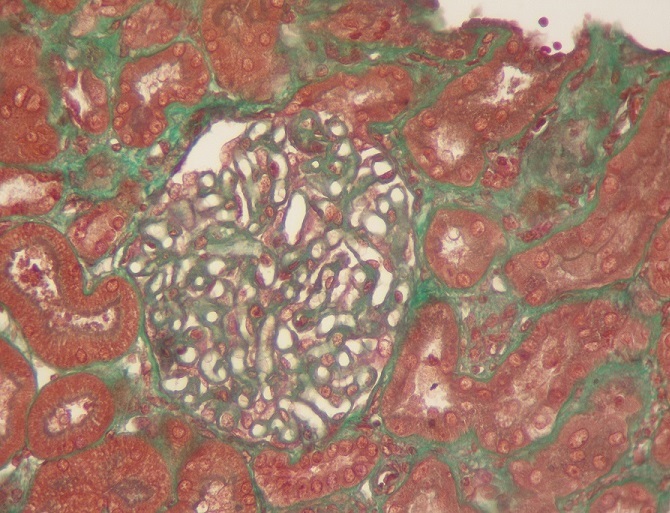
Trichrome de Masson (Gx100): épaississement régulier de la membrane basale glomérulaire sans prolifération endo ou extra membraneuse

**Figure 2:**
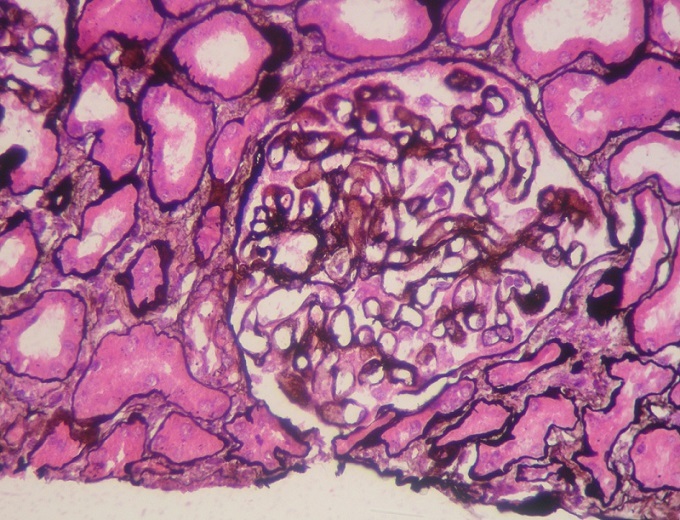
Coloration de la réticuline par imprégnation argentique (Gx100): épaississement régulier de la membrane basale glomérulaire sans prolifération endo ou extra membraneuse

Il s’agit d’une patiente âgée de 50 ans, sans antécédents pathologiques. En 2011, la patiente a présenté un syndrome oedémato-ascitique qui a conduit au diagnostic d’un syndrome néphrotique impur avec une albuminémie à 15g/l, une protidémie à 37g/l, une protéinurie évaluée à 7g/24h associée à une hématurie microscopique, une hypertension artérielle et une insuffisance rénale (créatininémie à 12 mg/l). Devant ce tableau une ponction-biopsie rénale a montré une glomérulonéphrite extra-membraneuse type II (Figure 1 et Figure 2) confirmée à l’immunofluorescence qui montrait des dépôts granuleux d'IgG et de C3 sur le versant externe de la membrane basale glomérulaire. Le bilan étiologique, comprenant un bilan d’auto-immunité, la recherche d’une néoplasie et un bilan infectieux bactérien et viral, était négatif confortant la nature idiopathique de cette glomérulopathie.

Par ailleurs, la patiente présentait une cytolyse avec une cholestase hépatique, dont les bilans morphologique et immunologique étaient négatifs. La ponction-biopsie hépatique retrouvait un aspect d’hépatite chronique active non-spécifique avec cholestase. Ce tableau hépatique a été alors mis sur le compte d’une toxicité médicameuteuse aux statines et fibrates. La cholestase et la cytolyse se sont amendées après arrêt de ces médicaments. Associé à ce tableau, le bilan retrouvait une anémie norcmochrome normocytaire à 6,4g/dl avec un VGM à 97 fl, arégénérative (taux de réticulocytes à 37900 éléments /mm^3^), les autres lignées étaient représentées normalement. La ferritinémie était à 245 ng/l et le dosage vitaminique (vitamine B12 et folates) était normal. La patiente ne présentait pas de syndrome inflammatoire (CRP à 1mg/l).

Le myélogramme montrait une moelle très riche, avec une hyperplasie de la lignée érythroblastique et une dysplasie multilignée sans excès de blastes. La coloration de Perls ne montrait pas de sidéroblastes en couronne. Le caryotype médullaire retrouvait un clone minoritaire avec une monosomie du chromosome 7 (six mitoses sur vingt). Le diagnostic d’un syndrome myélodysplasique de type anémie réfractaire avec dysplasie multilignée est retenu, avec risque intermédiaire 1 selon le score pronostique international des myélodysplasies. Un traitement selon le protocole Ponticelli est entrepris pendant six mois permettant l’obtention d’une rémission partielle du syndrome néphrotique. Sur le plan hématologique, la patiente est mise sous epoetin béta à la dose de 30000 UI/ semaine. A deux mois du début de l’érythropoïétine, la patiente n’est plus transfusée et a une hémoglobine à 9,8g/l.

## Discussion

L’association SMD et maladies auto-immunes est importante à connaitre. En effet, il est reporté que la survenue d’une MAI au cours de l’évolution d’un SMD aggrave le pronostic de ce dernier [4]. Les manifestations auto-immunes les plus fréquentes sont représentées par les vascularites [1]. Sur le plan physiopathologique, la survenue des MAI au cours des SMD serait déclenchée par l’excès d’apoptose des cellules hématopoïétiques et entretenue par des anomalies fonctionnelles des cellules dendritiques et des lymphocytes T [1].

La GEM est une maladie auto-immune à complexes immuns dont le diagnostic est histologique. Elle se caractérise par des dépôts granulaires d’IgG et de la fraction C3 du complément dans les parois des capillaires glomérulaires [5]. La GEM est idiopathique dans 80% des cas [6]. Les causes des GEM secondaires sont représentées par les infections à virus de l’hépatite B, la bilharziose et le plaudisme dans les pays en voie de développement et par l’atteinte lupique dans les pays développés. Elle peut s’associer également à des tumeurs solides. L’association glomérulonéphrite et SMD a été reporté dans la littérature (Tableau 1) [2-14] et la survenue d’une GEM au cours d’un SMD est exceptionnelle. Le tumor necrosis factor α (TNF-α) est impliqué dans la pathogénie de la GEM [7], en effet son expression est retrouvée dans les dépots extra-membraneux et dans les podocytes [7].

**Tableau 1:** Les associations syndrome myélodysplasique et glomérulopathies précédemment rapportés

Auteur	Glomérulonéphrite reportée	Nombre de cas
Saitoh et al. [2]	Prolifération mésangiale diffuse	1
	Syndrome néphrotique	4
Doukkali et al. [3]	GEM	1
Enright h et al. [4]	GEM	1
Paydas S. et al. [10]	GEM	1
Komatsuda A. et al. [11]	Glomérulonéphrite extra capillaire	1
Morschhauser F. et al. [12]	Glomérulonéphrite extra capillaire	1
	Amylose AL	1
Hayashi S. et al. [13]	Néphropathie à IgA	1
Hamzi MA et al. [14]	Glomérulonéphrite extra capillaire	1
Notre cas	GEM	1

Les associations syndrome myélodysplasique et glomérulopathies précédemment reportés

Cette cytokine est incriminée également dans l’apoptose accrue des cellules hématopoïétiques au cours des SMD [8]. Ces données indiquent que le TNF-α jouerait un rôle dans la genèse et l’association de ces deux pathologies. Il est suggéré aussi que l’excès d’apoptose dans les SMD libère des néo-antigènes suscitant une réponse immunitaire adaptative avec une dérégulation des fonctions des lymphocytes T-reg impliqués dans le maintien de la tolérance du soi [9]. Sur le plan phyisiopathologique, on n’arrive pas jusqu’à ce jour à expliquer de manière explicite l’association de ces deux pathologies. Mais il est clair qu’une dérégulation du système immunitaire dans les SMD favorise l’apparition de MAI.

## Conclusion

L’incidence des maladies auto-immunes est plus élevée chez les patients atteints de syndromes myélodysplasiques. L’atteinte rénale au cours de ces syndromes dont la physiopathologie est encore mal connue, reste rare ou plutôt sous-estimée. Ainsi, la recherche de manifestations néphrologiques, notamment glomérulaire, devrait faire partie du bilan initial des patients atteints de SMD.

## Conflits d’intérêts

Les auteurs ne déclarent aucun conflit d'intérêts.
